# Sinonasal microbiome remains stable during dupilumab therapy in chronic rhinosinusitis: a pilot study

**DOI:** 10.1007/s00405-026-10378-7

**Published:** 2026-06-15

**Authors:** T. Hirsch, U. Moser, A. Wolf, A. Andrianakis

**Affiliations:** Department of Otorhinolaryngology, Medical University of Graz, Graz, Austria

**Keywords:** Chronic rhinosinusitis, Dupilumab, Nasal microbiome, Alpha diversity, Beta diversity

## Abstract

**Purpose:**

This prospective pilot study aimed to evaluate whether dupilumab therapy induces measurable changes in the sinonasal microbiome of patients with chronic rhinosinusitis with nasal polyps (CRSwNP).

**Methods:**

Eight CRSwNP patients fulfilling criteria for biologic therapy were treated with dupilumab 300 mg biweekly for six months. Nasal swabs from the middle meatus were collected immediately before therapy initiation and after six months. Alpha diversity and beta diversity metrics were assessed, and differential abundance analysis was performed.

**Results:**

Dupilumab therapy did not result in significant changes in alpha diversity, beta diversity, or taxonomic composition. Minor, non-significant reductions in richness and Shannon diversity were observed. Principal coordinate analysis showed extensive overlap between pre- and post-treatment samples, supported by a non-significant PERMANOVA. No statistically significant differentially abundant taxa were detected. In contrast to the microbiome results, clinical outcomes improved markedly over the same period, with all patients showing a reduction in SNOT-22 of at least 12 points and a decrease in Nasal Polyp Score of at least 1 point.

**Conclusion:**

In this pilot CRSwNP cohort, no significant changes in sinonasal microbial diversity or composition were detected over six months of dupilumab therapy, despite marked clinical improvement. Larger, controlled studies are required to detect subtle or delayed microbial changes.

## Introduction

The role of the sinonasal microbiome in chronic rhinosinusitis with nasal polyps (CRSwNP) has emerged as an important focus, reshaping the understanding of this complex disease. Once thought to be caused primarily by bacterial infection in a sterile environment, CRS is now acknowledged as a condition strongly associated with microbial dysbiosis [1, 2]. Dysbiosis is characterized by reduced microbial diversity, altered proportions of key bacterial taxa, and the overgrowth of opportunistic pathogens [3–5]. Such disruptions may impair mucociliary clearance and immune regulation, promoting inflammation, epithelial damage, and disease progression. Furthermore, decreased diversity and the proliferation of biofilm forming bacteria have been linked to symptom severity, treatment resistance, and recurrent disease [2, 6].

In recent years, the introduction of monoclonal antibody therapies has expanded treatment options for patients with CRSwNP. Dupilumab, a human monoclonal antibody, targets the interleukin-4 receptor alpha subunit, thereby inhibiting the Th2 prominent IL-4 and IL-13 signaling. While studies on microbiome changes after FESS show considerable interindividual variability, research into the impact of dupilumab on the sinus microbiome is limited.

The primary aim of this study was to investigate whether the microbiome of the middle nasal meatus changes in response to biologic therapy with dupilumab. This work aims to deepen the understanding of how pharmacological interventions influence the sinonasal microbiome in CRSwNP.

## Materials and methods

Ethical approval was obtained from the institutional ethics committee, and written informed consent was obtained from all participants.

Patients with uncontrolled CRSwNP who fulfilled EPOS2020 criteria for biologic therapy [1] were treated with dupilumab 300 mg subcutaneously every 2 weeks for six months. All included patients had previously undergone endoscopic sinus surgery (ESS) prior to initiation of biologic therapy. Throughout the entire six-month observation period, all patients continued their standard-of-care intranasal corticosteroid therapy. No patient received antibiotics or systemic corticosteroids during the six-month study period.

Nasal swabs were collected from the middle nasal meatus immediately before therapy initiation (time_of_sample 3) and again after six months of treatment (time_of_sample 4). Samples were stored at –80 °C until further analysis. DNA extraction was performed using the DNeasy® PowerSoil® Pro Kit. Quantitative real-time PCR was performed to determine the total bacterial load in each sample. Sequencing data were processed using the QIIME2 pipeline [7] with taxonomic assignment based on the SILVA 138 database [8]. Potential contaminants were removed using the prevalence-based method of the decontam R package [9].

Data were normalized using scaling with ranked subsampling (SRS). Alpha diversity (Shannon index, richness) and beta diversity (Bray–Curtis dissimilarity) were calculated based on the normalized ASV table. Statistical analyses and visualizations were performed in R using MicrobiomeExplorer [10] and Namco [11].

## Results

### Clinical outcome

8 patients were included in the study. Baseline patient characteristics are presented in Table 1. Clinical outcomes improved consistently in all patients following dupilumab therapy. Each patient experienced a reduction of at least 20 points in the SNOT-22 score, exceeding the threshold for clinically significant improvement of ≥ 12 points. Similarly, the NPS decreased by 2 points or more in all cases, surpassing the clinically relevant cutoff of ≥ 1 point. These uniform improvements highlight the clinical effectiveness of dupilumab in this cohort. Individual patient data are presented in Table 2.

**Table 1:** Baseline patient characteristics

Age, years	Min–Max	**25–67**
	Mean (SD)	**40 (14.1)**
Sex, n (%)	Male	**6 (75%)**
	Female	**2 (25%)**
Type 2 inflammatory markers	Blood eosinophils (G/L), mean (SD)	**0.58 (0.3)**
	IgE in serum (U/mL), mean (SD)	**208.59 (244.81)**
Comorbidities, n (%)	Asthma	**6 (75%)**
	Allergy	**7 (87.5%)**
	Smoking	**1 (12.5%)**
Prior ESS, n (%)	1	**3 (37.5%)**
	2	**1 (12.5%)**
	3	**1 (12.5%)**
	4	**2 (25%)**
	5	**1 (12.5%)**
Clinical scores, mean (SD)	SNOT-22 (0–110)	**55.9 (21.5)**
	NPS (0–8)	**4,8 (1.0)**

*ESS* Endoscopic Sinus Surgery, *IgE* Immunoglobulin E, *SNOT-22* Sino-Nasal Outcome Test 22, *NPS* Nasal Polyp Score

**Table 2:** Clinical outcome

Patient	SNOT-22 pre	SNOT-22 post	NPS pre	NPS post
1	68	9	5	1
2	58	38	5	1
3	69	8	4	1
4	63	35	4	1
5	86	33	5	1
6	29	2	6	2
7	53	12	3	1
8	21	9	6	2
**Mean**	**55.9**	**18.3**	**4.8**	**1.3**

*SNOT-22* Sino-Nasal Outcome Test 22, *NPS* Nasal Polyp Score

## Microbiome outcome

**Fig. 1:**
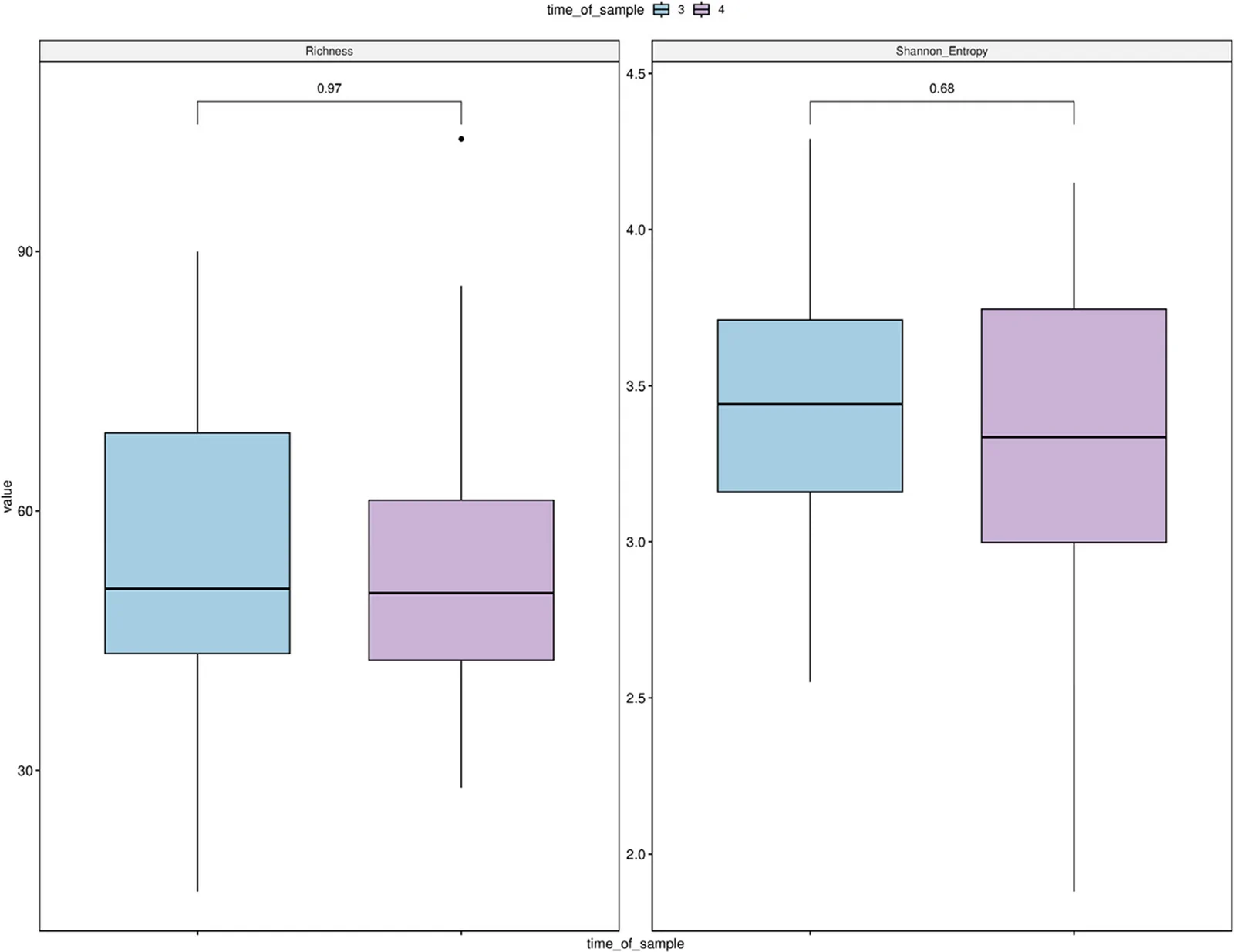
Alpha diversity before and after dupilumab therapy. Left: richness (observed ASVs). Right: Shannon index. “Time of sample 3” = pre-treatment; “time of sample 4” = post-treatment. Boxplots show median, interquartile range, distribution, and outliers; brackets indicate group comparisons with *p*-values

Alpha diversity metrics in Fig. 1 showed no significant changes in the sinonasal microbiome before and after dupilumab therapy. Both richness and Shannon diversity remained stable between pre-treatment (time_of_sample 3) and post-treatment (time_of_sample 4), with no evidence of systematic increases or decreases.

The statistical comparison yielded p = 0.97 for richness and p = 0.68 for the Shannon index, confirming the absence of detectable alpha-diversity shifts during the treatment period.

**Fig. 2:**
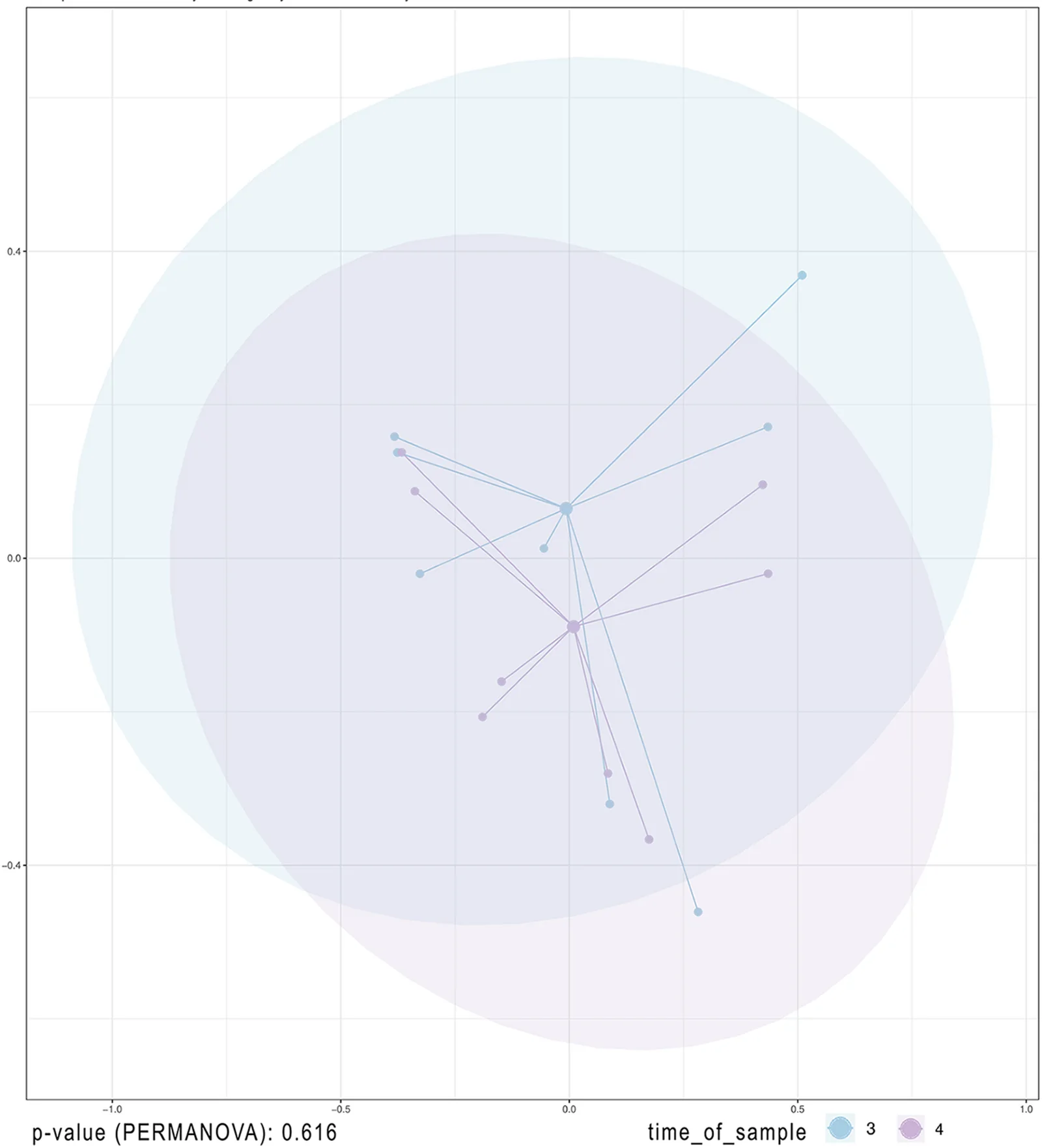
Beta diversity before and after dupilumab therapy based on Bray–Curtis dissimilarity. PCoA shows no distinct clustering between samples collected at time point 3 (pre-treatment) and time point 4 (post-treatment). PERMANOVA p = 0.616

Beta diversity analysis shown in Fig. 2 using Bray–Curtis dissimilarity showed substantial overlap between pre- and post-dupilumab samples. The PCoA plot demonstrated no separation between time_of_sample 3 (pre-treatment) and time_of_sample 4 (post-treatment), and paired trajectories indicated that individual microbiome profiles largely remained the same over time.

This was confirmed by PERMANOVA (p = 0.616), indicating that dupilumab therapy did not lead to detectable shifts in overall community structure.

**Fig. 3:**
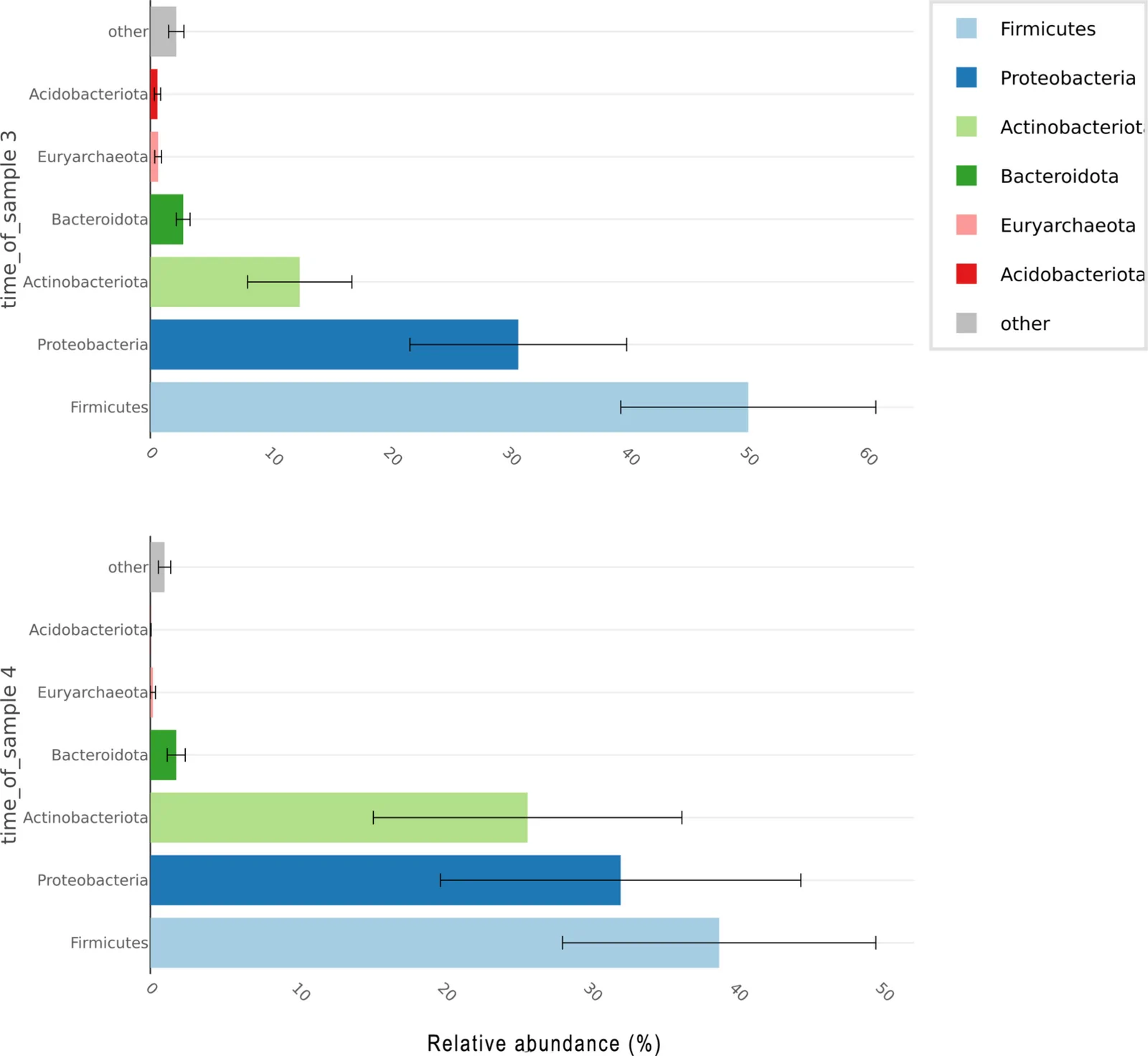
Relative abundance of the six most common bacterial phyla in sinunasal samples before (time of sample 3) and after (time of sample 4) dupilumab therapy. Bars represent mean ± standard error for each phylum

The results also line up with the Bar Chart in Fig. 3 showing a relatively stable microbiome structure. The relative abundance profiles at the phylum level were comparable between both time points. Firmicutes and Proteobacteria continued to dominate the sinonasal microbiome at both time points, with mean abundances showing only minor, non-systematic fluctuations. Actinobacteriota displayed a small increase post-treatment, while Bacteroidota, Euryarchaeota, Acidobacteriota and the remaining low-abundance groups (“other”) showed only minimal variation. These shifts did not reflect any consistent directional pattern and therefore do not indicate a treatment-related restructuring of the microbiome.

**Fig. 4:**
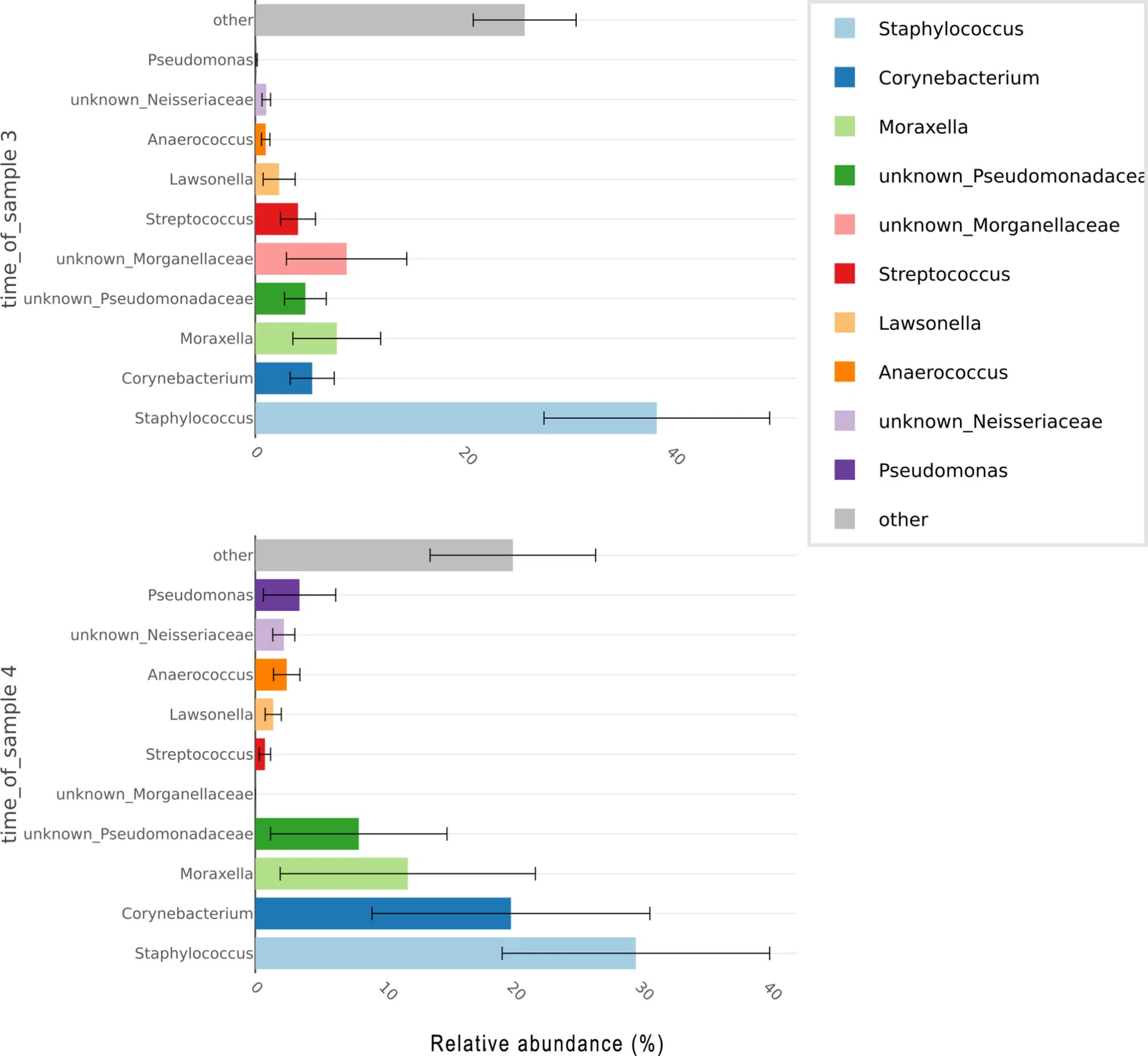
Relative abundance of the ten most common bacterial genera in sinunasal samples before (time of sample 3) and after (time of sample 4) dupilumab therapy. Bars show mean percentages with standard errors

At the genus level, the overall community structure remained stable, although several taxa showed modest but non-significant shifts (Fig. 4). *Staphylococcus* decreased after treatment, while *Corynebacterium*, *Moraxella*, and members of the unknown_Pseudomonadaceae group exhibited moderate increases at time_of_sample 4. Minor fluctuations were observed in *Streptococcus*, *Anaerococcus*, *Lawsonella*, and low-abundance genera, but none reached statistical significance. These patterns again indicate no consistent treatment-related restructuring of the sinonasal microbiome.

Differential abundance analysis using DESeq2, performed across multiple taxonomic levels, did not identify any statistically significant taxa associated with the pre- or post-treatment state. No unadjusted p-values fell below 0.05.

## Discussion

The primary aim of this pilot study was to determine whether treatment with dupilumab produces measurable changes in the nasal microbiome of patients with CRS. Although CRS is strongly associated with dysbiosis, no significant alterations in microbial composition, alpha diversity, or beta diversity were detected after six months of therapy. These findings must be interpreted within the broader context of microbiome research in CRS, which consistently shows that CRS patients differ markedly from healthy individuals in terms of microbial structure and stability [12–14].

Previous studies have demonstrated that CRS is associated with reduced microbial diversity and altered community composition compared with healthy sinonasal microbiomes, including compositional changes or an increased burden of pathogenic taxa [12–15]. Reduced diversity can impair resilience, enabling overgrowth of taxa such as *Staphylococcus aureus*, which is closely associated with increased inflammation, biofilm formation, and disease persistence [12].

Importantly, recent studies report that dupilumab therapy does not induce significant changes in the nasal microbiome, with stable alpha and beta diversity observed despite clinical improvement or changes in cytologic markers [16, 17].

In addition, Ryser et al. recently reported that dupilumab treatment in patients with diffuse type-2 chronic rhinosinusitis was associated with clear clinical improvements and a shift toward a “health-associated” nasal microbiome profile. This shift was mainly characterized by a reduction in *Staphylococcus aureus* burden and an increase in commensal taxa, although the authors emphasized that these changes were not uniform across all participants and showed marked interindividual variability [18].

Even though all patients experienced clinically significant improvements in both symptom burden and polyp size, defined by a reduction in SNOT-22 of at least 12 points and a decrease in NPS of at least 1, these clinical improvements were not matched by detectable changes in the sinonasal microbiome.

While modest fluctuations in genera such as *Staphylococcus*, *Corynebacterium*, *Dolosigranulum*, and *Moraxella* were noted, none reached statistical significance. This supports the interpretation that dupilumab acts primarily through immunologic mechanisms rather than through direct effects on microbial community structure.

Together, these findings highlight that biologic-associated microbiome shifts may occur in some individuals but are neither universal nor robust, and may depend on disease phenotype, baseline colonization patterns, and microbial biomass.

The stability of the microbiome may be explained by the persistence of biofilm-forming bacteria, which can remain in the sinonasal environment despite changes in immune activity [3, 19, 20]. Furthermore, the interaction between the microbiome and host immune response is complex; CRS may be driven as much by inappropriate immune reactivity to commensals as by overt pathogenic colonization [12, 21, 22]. Studies investigating microbiome directed therapies, such as probiotics, have shown mixed results in CRS and underscore that modifying the sinonasal microbiome remains challenging. [23].

Several limitations must be considered when interpreting these results. First, this was a small pilot study including only eight patients and no control group, which limits both internal validity and generalizability. Second, the analysis was underpowered to detect subtle differences in microbiome diversity or differential abundance, particularly in the context of low microbial biomass that is typical for sinonasal sampling. The present negative findings should therefore be interpreted as an absence of detectable changes rather than evidence that dupilumab has no effect on the sinonasal microbiome. Finally, sampling from a single sinonasal site at only two time points may have not captured short-term temporal fluctuations in the sinonasal microbiome.

In conclusion, our study reinforces an emerging pattern in the literature: Six months of Dupilumab therapy in patients with CRS led to significant clinical improvements, but no measurable changes in sinonasal microbiome diversity, or taxonomic composition were detected. Future, adequately-powered studies including a larger sample size, and appropriate control group are needed to clarify whether subtle, delayed or patient-specific changes in the sinonasal microbiome occur during biologic treatment of CRS.

## Data Availability

The datasets generated and analyzed during the current study are available from the corresponding author on reasonable request.
